# The *Retrovirology* open access experience

**DOI:** 10.1186/1742-4690-8-S2-O26

**Published:** 2011-10-03

**Authors:** Kuan-Teh Jeang

**Affiliations:** 1 Editor-in-Chief, Retrovirology

## 

The Open Access (OA) model of publishing has revolutionized the egalitarian distribution of knowledge, especially to those who are least able financially to pay for subscription access. Because OA journals are newer ventures, they face several challenges. They often lack the track record and established reputation necessary to attract the best quality submissions. In general, most OA journals also do not sport the high Impact Factors numbers that many scientists consider to be crucial for advancing their careers. How then does one go about starting and continuing a successful OA journal? I have written briefly in the past about *Retrovirology's* Open Access experience [1] and the process that we go through to develop recognition [2] for our authors and citation and visibility [3] for *Retrovirology* papers. Here, I will discuss some of these issues in greater depth and touch on the realities and perceptions about OA journals.
